# ELDER MISTREATMENT, MORTALITY, AND HOSPITAL READMISSION AMONG MEDICARE BENEFICIARIES, 2015–2018

**DOI:** 10.1093/geroni/igac059.1671

**Published:** 2022-12-20

**Authors:** Monique Pappadis, Leila Wood, Allen Haas, Yong-Fang Kuo, Charles Mouton

**Affiliations:** University of Texas Medical Branch, Galveston, Texas, United States; University of Texas Medical Branch at Galveston, Galveston, Texas, United States; University of Texas Medical Branch at Galveston, Galveston, Texas, United States; University of Texas Medical Branch at Galveston, Galveston, Texas, United States; University of Texas Medical Branch at Galveston, Galveston, Texas, United States

## Abstract

Elder mistreatment (EM) is a growing public health and safety crisis, with long-term consequences for individuals, families, and communities. We explored whether older adults hospitalized with a primary diagnosis of EM was associated with an increased risk of mortality and unplanned hospital readmission compared to those with a secondary EM diagnosis. We further examined whether EM type and hospital setting was associated with risk of mortality and unplanned hospital readmission. Using 100% of 2015-2018 Medicare files of hospitalized Medicare Fee-for-Service beneficiaries aged 66 and over, we used Kaplan-Meier and Cox proportional hazard models to estimate mortality and unplanned readmission rates by primary versus secondary EM diagnosis, EM type, and facility type. 11,023 patients were hospitalized with an EM diagnosis. The majority were female (64.1%) and Non-Hispanic/Latinx White (74.3%). Neglect was the most common EM type. The three-year mortality rate was 56.7% and one-year readmission rate was 53.8%. Compared to other EM types, patients diagnosed with neglect had a 2.20 (95% Confidence Interval [CI]=1.88-2.56) and 3.21 (95% CI=2.32-4.43) times greater risk for mortality within and after 50-days from discharge, respectively. Patients discharged from a skilled nursing facility (SNF) were at an increased risk of mortality and unplanned readmission compared to those discharged from an acute hospital. Hospitalized patients with a primary EM diagnosis were associated with an increased risk of mortality and readmission compared to those with a secondary diagnosis. Future work should explore care patterns before and after EM diagnosis to identify potential time points for medical and social intervention.

